# SeqTrim: a high-throughput pipeline for pre-processing any type of sequence read

**DOI:** 10.1186/1471-2105-11-38

**Published:** 2010-01-20

**Authors:** Juan Falgueras, Antonio J Lara, Noé Fernández-Pozo, Francisco R Cantón, Guillermo Pérez-Trabado, M Gonzalo Claros

**Affiliations:** 1 Departamento de Lenguajes y Ciencias de la Computación, Universidad de Málaga, Málaga, Spain; 2 Plataforma Andaluza de Bioinformática, Universidad de Málaga, 29071 Málaga, Spain; 3 Departamento de Biología Molecular y Bioquímica, Universidad de Málaga, 29071 Málaga, Spain; 4 Departamento de Arquitectura de Computadores, Universidad de Málaga, Málaga, Spain

## Abstract

**Background:**

High-throughput automated sequencing has enabled an exponential growth rate of sequencing data. This requires increasing sequence quality and reliability in order to avoid database contamination with artefactual sequences. The arrival of pyrosequencing enhances this problem and necessitates customisable pre-processing algorithms.

**Results:**

SeqTrim has been implemented both as a Web and as a standalone command line application. Already-published and newly-designed algorithms have been included to identify sequence inserts, to remove low quality, vector, adaptor, low complexity and contaminant sequences, and to detect chimeric reads. The availability of several input and output formats allows its inclusion in sequence processing workflows. Due to its specific algorithms, SeqTrim outperforms other pre-processors implemented as Web services or standalone applications. It performs equally well with sequences from EST libraries, SSH libraries, genomic DNA libraries and pyrosequencing reads and does not lead to over-trimming.

**Conclusions:**

SeqTrim is an efficient pipeline designed for pre-processing of any type of sequence read, including next-generation sequencing. It is easily configurable and provides a friendly interface that allows users to know what happened with sequences at every pre-processing stage, and to verify pre-processing of an individual sequence if desired. The recommended pipeline reveals more information about each sequence than previously described pre-processors and can discard more sequencing or experimental artefacts.

## Background

Sequencing projects and Expressed Sequence Tags (ESTs) are essential for gene discovery, mapping, functional genomics and for future efforts in genome annotations, which include identification of novel genes, gene location, polymorphisms and even intron-exon boundaries. The availability of high-throughput automated sequencing has enabled an exponential growth rate of sequence data, although not always with the desired quality. This exponential growth is enhanced by the so called "next-generation sequencing", and efforts have to be made in order to increase the quality and reliability of sequences incorporated into databases: up to 0.4% of sequences in nucleotide databases contain contaminant sequences [1,2]. The situation is even worse in the EST databases, where vector contamination rate reach 1.63% of sequences [3]. Hence, improved and user friendly bioinformatic tools are required to produce more reliable high-throughput pre-processing methods.

Pre-processing includes filtering of low-quality sequences, identification of specific features (such as poly-A or poly-T tails, terminal transferase tails, and adaptors), removal of contaminant sequences (from vector to any other artefacts) and trimming the undesired segments. There are some bioinformatic tools that can accomplish individual pre-processing aspects (e.g. TrimSeq, TrimEST, VectorStrip, VecScreen, ESTPrep [4], crossmatch, Figaro [5]), and other programs that cope with the complete pre-processing pipeline such as PreGap4 [6] or the broadly used tools Lucy [7,8] and SeqClean [9]. Most of these require installation, are difficult to configure, environment-specific, or focused on specific needs (like a design only for ESTs), or require a change in implementation and design of either the program or the protocols within the laboratory itself. Moreover, it is not always possible to connect them easily with further processing tools for annotation or assembling. There are Web implementations (ESTAnnotator [10], ESTpass [11] or ESTExplorer [12]) that start with pre-processing and end with assembling and/or annotating ESTs, but no Web page is devoted exclusively to pre-processing. Further, these implementations are focused more on annotation than on a correct pre-processing, and tend to disregard the fact that poorly pre-processed sequences will produce effectively useless annotations.

This paper describes SeqTrim, a software tool containing a flexible pipeline that successfully deals with pre-processing of any sequence read. Its performance is compared with other broadly used applications, and when using high-throughput datasets.

## Implementation

SeqTrim has been programmed in Perl 5.8 using BioPerl libraries, can be executed as a command line tool or as a Web tool http://www.scbi.uma.es/seqtrim, and tasks are queued to a HP-SuperDome computer. The command line version is more suitable for automatic batch processing, workflows, or high-throughput analyses, while the Web interface is more appropriate for user interactivity. It makes use of the external programs phred [13,14] for obtaining sequence and quality values, BLAST to compare sequences, and RepeatMasker to mask repetitions and low complexity regions. This will work in any unix/linux release, including OSX.

Provided that the dependencies are installed, uncompressing SeqTrim in /usr/local (or in any other directory defined in the $PATH) is sufficient to make it operable. Configuration parameters in the seqtrim directory are customisable by the user, transiently or permanently: working parameters can be permanently modified by editing the 'seqtrim.conf', or changed for a single run via command-line options or the Web interface. The seqtrim directory also contains the necessary databases, an editable file called 'RE_sites.txt' that contains the usable restriction sites, and another editable file named 'adaptorSeqs.txt' which contains a list of default adaptor sequences. Database modification is achieved simply by adding or removing sequences in FASTA format in the seqtrim/DB directory. Before each execution, SeqTrim verifies if something has been added to databases for incorporation of new sequences thereafter.

**Figure 1:**
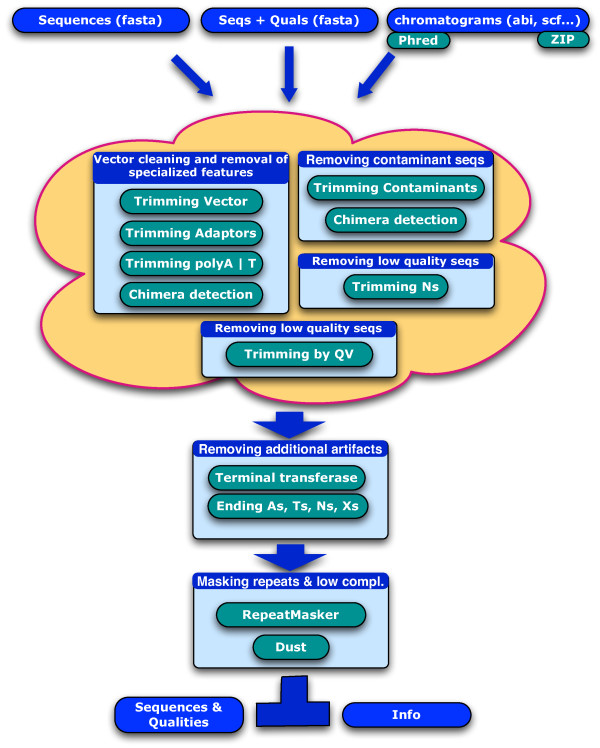
**Detailed data-flow diagram of the SeqTrim pipeline**. It consists of four major steps (vector cleaning and removal of specialised features, two quality trimming steps, and contamination removal) that can be executed in any order or skipped, and two ending steps (artefact removal, and low complexity and repeat masking). The output is stored in a private area defined by an e-mail address, and can be looked up asynchronously.

The pipeline underlying SeqTrim runs through four independent and interchangeable processes (vector and other specialised features removal, quality trimming, indetermination trimming, and contamination removal) plus two optional ending steps (artefact removal, and low complexity and repeat masking) (Fig. 1). One of the main strengths of SeqTrim is that, even if a default pipeline order is provided, users can change the flow completely, or can skip one or more steps.

Execution time for a single sequence will depend on the complexity of the given sequence. For example, SeqTrim (without masking with RepeatMasker) takes 0.304 s/read in a 2.2 GHz Intel Core 2 duo processor and 0.477 s/read in a 1.6 GHz Itanium 2 processor when analysing 96 EST reads (a complete micro-plate) with an average length of 755 nt. When it has been tested (without the need for vector cleaning nor removal of other specialised features) with 1000 GS-FLX pyrosequencing reads with an average length of 236 nt, execution takes 0.074 s/read and 0.083 s/read, respectively, in the processors mentioned above. A true high-throughput use of SeqTrim must consider use of the command line version, since the Web interface facet becomes unsatisfactory when showing jobs with more than 10,000 reads; the browser taking 15 s to reply to a single click. However, the SeqTrim Web server is able to process up to 40,000 reads without "hanging", Safari always reacting faster than Firefox.

## Algorithm

The recommended SeqTrim pipeline starts with vector detection using vector libraries and the removal of special features. The next step involves to trimming low quality 5'- and 3'-tails. Finally, any sequence coming from a contaminating source is removed. Two optional end steps are focused on removing any other experimental artefacts arising from molecular modifications and the masking of low complexity regions.

### Input and output

SeqTrim accepts usual sequence formats: FASTA file(s) with or without the corresponding quality value file, phd format file from phred, and chromatograms, all of them with any number of sequences and optionally compressed in zip format. Text files are directly processed, but when input sequences are chromatograms, the external program phred is employed to obtain the quality value file. It must be understood that phred's low quality trimming option is disabled for such a conversion. Since the first word in the description line of every input sequence is considered its identifier, checks for sequence name duplications, as well as consistency between the sequence file and the quality value file (if provided), are performed.

**Figure 2:**
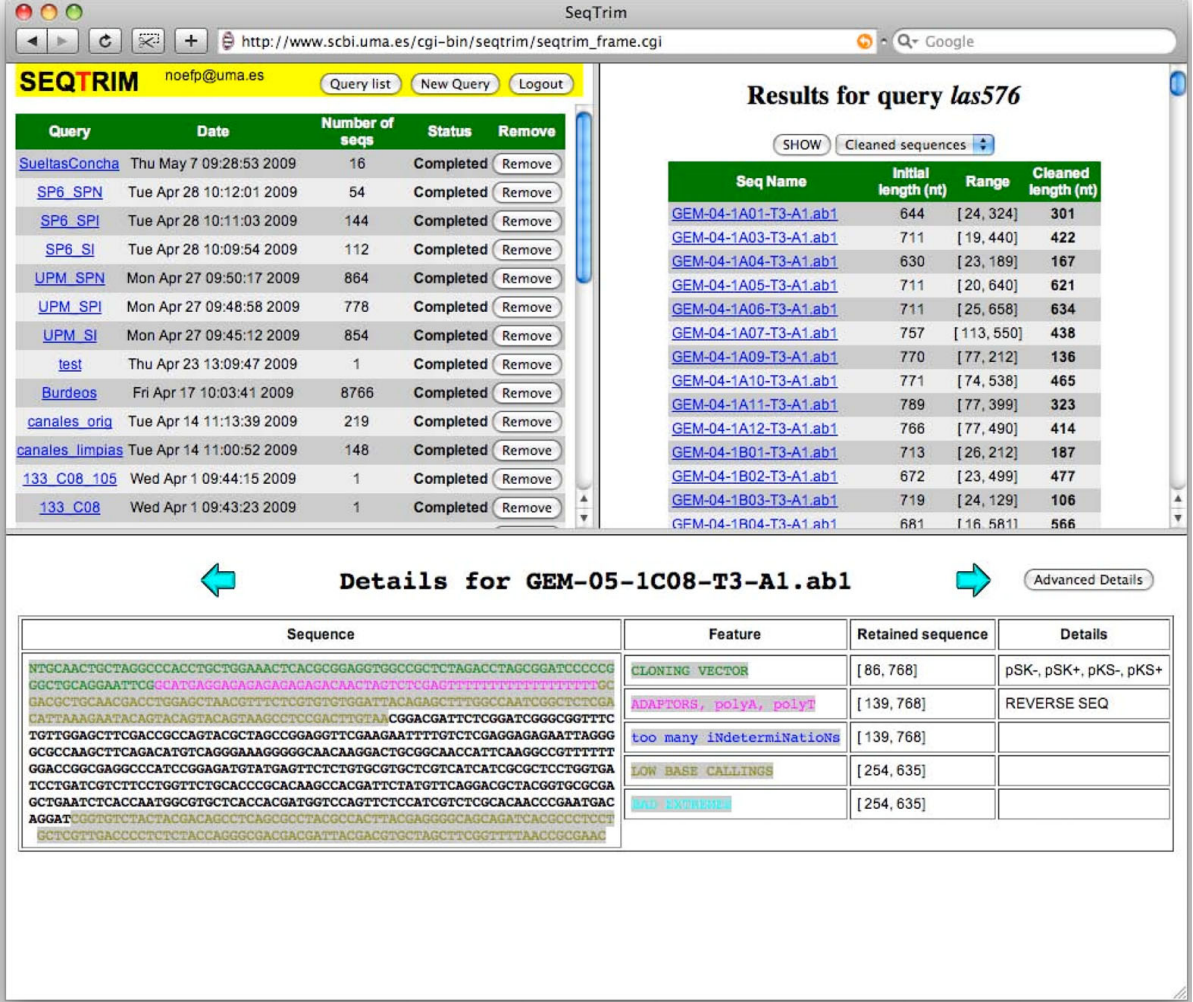
**Example of sequence trimming using different sets of sequences**. The upper-left frame displays the status of all executions of SeqTrim made by the user ordered by date and time of run. The upper-right frame displays the list of the sequences analysed in a single query with original length, and range and length of the trimmed sequence. Buttons for saving result files and to obtain parameters and execution order are displayed at the end of the sequence list. The bottom frame shows the original sequence coloured according to the different segments that have been removed. Legend on the right explains colour codes and a button to ask for more details are presented. Sequences to be detailed in the bottom frame are the same as listed in the upper-right frame.

Several output formats (like a FASTA file containing only trimmed inserts, a text file containing user-readable information concerning the trimming events for each sequence, a text file containing the names of the rejected sequences, or a FASTA file with masked sequences) can be obtained either from the Web interface or the command line. Nucleotides whose quality value (QV) is not greater than 20 (by default) are changed to lowercase. A coloured output of each sequence can also be seen on the screen, either using the command line or the Web interface (Fig. 2), which is intended to help users in the evaluation of pre-processing results. Results will be stored for at least one month in the Web server using an e-mail address as identifier since no account is needed for SeqTrim usage.

### Vector cleaning and removal of specialised features

The recommended first pipeline step starts by detection of cloning vector by means of NCBI's UniVec and the EMBL's emvec vector/adaptor libraries using BLAST with relaxed parameters (q -5, G 3, E 3, F 'm D', e 1e-10) to account for higher error rates at the beginning and end of reads. Users do not need then to specify the cloning vector. BLAST alignment is parsed to identify regions that correspond to vector sequences, even if these regions are spliced into smaller DNA fragments that match in opposite orientations. SeqTrim is designed to locate cloning restriction sites only when cloning vector was not identified. Cloning restriction sites must be entered in the parameters panel of SeqTrim.

Next is location of special features [adaptors, poly-A tails (only for ESTs) and poly-T tails (only for ESTs, which indicates that the sequence is in the reverse orientation)] appearing in many sequences. Special features must be removed since they (i) provide false sequences, (ii) mislead assembling or clustering algorithms that can be further used with these sequences, and (iii) mislead researchers who use these contaminated sequences. Adaptors are located with BLAST2SEQ, customising its parameters for short sequences (W 7, F 'F', program 'blastn'). Poly-A and poly-T tails are detected by the function findPolyATs (Appendix) developed by the authors, which includes the removal of one or more A's at the 3' end of a sequence. The algorithm has been set to produce maximal sensitivity while maintaining a very strict rule for false positives: sequences that are not compliant with all necessary criteria are always rejected. In the case of ESTs, insert orientation is detected by the presence of poly-A or T tails, and chimeric inserts are determined by the concomitant presence of two of these tails in the same sequence. Poly-A or poly-T detection is skipped if input sequence does not come from a cDNA, in order to gain CPU time. In the parameters panel, users can specify adaptors, DNA source (genomic or cDNA) and orientation (if known) of inserts.

### Quality trimming

A sequence read can contain bases of very low quality, which can mislead further processing. Therefore, the base-calling quality assessment for each nucleotide is taken into account to trim the original sequence, in order to obtain the longest sequence with the highest quality. In cases where the input sequences are in a text FASTA format and low quality nucleotides are expressed by N's (indetermination), SeqTrim can extract the largest subsequence containing less than 18% of indetermination. Values for assessing sequence quality can be changed in the parameter panel. Since not all sequences include N's, quality trimming is split into two independent steps (Fig. 1) in order to enable users to skip the useless function. Trimming by QV is automatically skipped when input sequences are not chromatograms or a quality value file is not entered. Stringency of QV trimming can also be changed by means of the parameter panel.

### Removal of contaminant sequences

During the experimental process, cloned sequences can result from contamination sources such as DNA from the *Escherichia coli *genome, cloning vector, cell plasmids, organelles, viruses, yeast, or humans (due to handling). Screening of contaminant sequences is based on BLAST comparisons with trimmed sequences against a database of likely contaminants using default parameters and an expected cutoff fixed to 1e-3 and a minimal score of 60. The BLAST result is analysed by the in-house algorithm FindContaminantSeqs (Appendix) to establish when hits are real contaminations. Users can vary the stringency of this analysis by changing the minimal length of sequence considered as a significant contaminant in the parameter panel. SeqTrim is distributed with the genomes of *E. coli*, *Saccharomyces cerevisiae*, lambda phage and several mitochondria. More databases can be added (or removed) by the user. Length, position and identity of the contaminant sequence are returned for user information. The vector database is re-screened again at this moment, as well as adaptors, which serves to identify putative chimeras.

### Removal of other artefacts

This optional step is intended to be performed at the end of pre-processing, since it is focused on removing any experimental artefacts introduced in the apparently cleaned insert that are owing to molecular modifications. For example, extensions introduced by the terminal transferase enzyme, the N's and/or X's at both ends, and the T's from the 5'-end and/or A's from the 3'-end are discarded here. These removals are accomplished by means of the in-house function Look_for_artefacts (see Appendix).

### Masking low complexity regions and repeats

This is the last step of the SeqTrim pipeline, in which the unwanted sequence is not removed but masked, since low complexity regions and repeats are part of the real sequence, even if they can mislead further computer analysis. Low complexity regions due to simple nucleotide repeats are masked by the in-house function LowComplexityMasking (see Appendix). Repeats in nucleotide sequences are masked by employing RepeatMasker using species-specific repeat libraries obtained from the RepBase [15]. The searching algorithm for RepeatMasker has been fixed to WU-BLAST in order to reduce the time spent in one sequence analysis.

### Rejection criteria

A pre-processed sequence is finally rejected if it complies with one the following criteria: (1) there is no insert between identified cloning vector boundaries; (2) the usable sequence is not long enough (less than 100 bp by default, which can be changed in the parameter panel); (3) there are possibly two inserts (chimeric inserts are determined by the presence of two poly-A/T tails, or detection of an internal adaptor and/or cloning restriction site); (4) the whole insert was masked. In contrast to other pre-processing algorithms, absence of cloning vector is not a reason for rejection since sometimes useful sequence starts beyond vector boundaries, or there can exist vector rearrangements that make it unrecognisable although the insert is preserved (results not shown).

## Results

Developer versions of SeqTrim have been used for some years at Málaga University using actual data from various previous studies such as ESTs from xylem tissue of *Pinus pinaster *[16], ESTs from photosynthetic tissues of *Pinus sylvestris *(C Avila *et al.*, unpublished results), SSH gene libraries from pine (F.R. Cantón *et al.*, manuscript in preparation), or assembling BAC sequences (M. G. Claros *et al.*, unpublished results). Now, a collection of comparative analyses are able to demonstrate that SeqTrim outperforms other pre-processing software and that it is able to handle huge amounts of sequence.

### Comparison with other algorithms

SeqTrim performance has been compared to other widely used pre-processors such as SeqClean (which acts similarly to SeqTrim), Lucy2 [8] (which makes use of several base caller algorithms and additional specific algorithms) and ESTPrep [4] (which makes use of a heuristic match function to detect sequence features, and phred to obtain quality values). Although cross-match is a restricted Smith-Waterman algorithm and has been incorporated into some EST processing packages, it has been discarded because it does not remove but masks vector-like regions, takes too much time to execute, and is not better than SeqClean or Lucy [3]. Since ESTs are the only kind of sequence that can be used in all programs, a collection of 576 EST chromatograms obtained in our laboratory [16] was used as the testing sequence set. These reads resulted in 438,550 nucleotides, of which 53.8% were considered insert by ESTPrep, 53.6% by SeqClean, 37.4% by Lucy and 31.74% by SeqTrim. The sequence reads had an average length of 761 nucleotides but, once pre-processed, the average insert size was 569 for ESTPrep, 562 for SeqClean, 490 for Lucy and 409 for SeqTrim. Both kinds of data clearly show that SeqTrim renders the shortest sequences; in fact, SeqTrim provides the shortest final sequence in 218 cases, the second-shortest in 19 cases, the third-shortest in only two cases, and never provides the longest sequence.

Even with shorter sequences, SeqTrim is able to retain more information about trimmed sequences than the other programs (Fig. 2). With the default pipeline, SeqTrim is able to detect (Fig. 2) the presence of cloning vector at the 5'-end and the existence of a poly-T segment, which indicates that this sequence was cloned in reverse. Hence, SeqTrim can return a reverse complement of such a sequence in order to acquire it in the same orientation as the others. If quality trimming had been performed in the first instance, the poly-T would have been removed and the researcher would have recovered a trimmed sequence in the correct orientation. As can be seen in the details of Fig. 2, the user is informed that this sequence was reversed.

**Figure 3:**
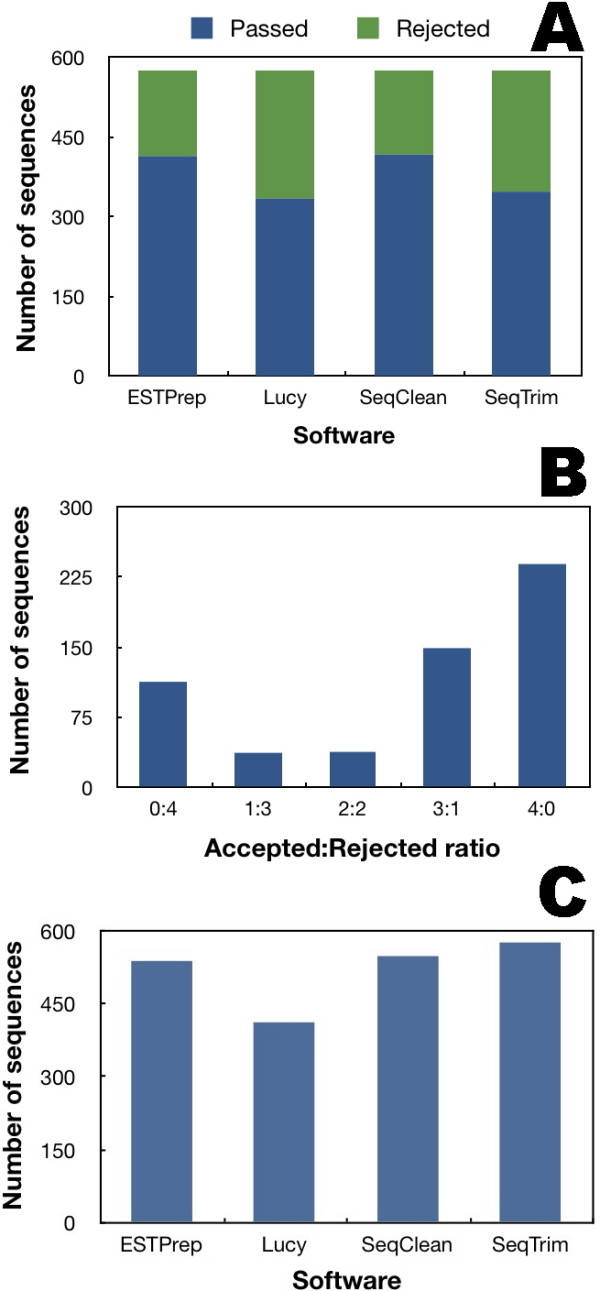
**Performance comparison of SeqTrim, SeqClean, Lucy and ESTPrep**. A, Overview of rates of sequence acceptance and rejection by each tested algorithm. B, Degree of agreement from concordance in rejection (0:4, which means none of the four implementations accept the same sequence) to concordance in acceptance (4:0, which means that the four accept the same sequence). C, Degree of agreement of SeqTrim with respect to the other three programs.

With regard to the number of passed/rejected sequences (Fig. 3), ESTPrep validates 415, Lucy 335, SeqClean 418 and SeqTrim 348. Lucy is therefore the most restrictive, with SeqTrim nearly as restrictive, and SeqClean and ESTPrep each returning a similar result and being the most permissive. Equivalent outcomes were also derived when assessing the number of sequences instead of the number of nucleotides. Concordance among the algorithms was tested by assessing the number of sequences accepted and rejected. The four software programs agree in 352 sequences (113 rejected by all and 239 accepted by all, Fig. 3B), which correspond to 61.1% of sequences. If agreement is relaxed to three coincidences, the concordance rate increases to 93.4%. SeqTrim is primarily consistent with ESTPrep and SeqClean (93.4% and 95.1%, respectively, Fig. 3C), with ESTPrep and SeqClean showing only slightly less consistency between them (92.0%). SeqClean ist not as consistent with Lucy (71.5%), an Lucy disagrees similarly with ESTPrep and SeqClean (70.5% and 72.4%, respectively).

Agreement between the most coincident softwares (SeqTrim and SeqClean) was cross-verified. When sequences trimmed by SeqTrim were entered into SeqClean, no changes were observed, indicating that both items of software remove the same features. On the contrary, when sequences trimmed by SeqClean were entered into SeqTrim, most of them were slightly shorter mainly due to adaptor removal that SeqClean did not detect, although sometimes differences were related to low quality sequences, or chimeric sequences, that were not removed by SeqClean (results not shown).

### Trimming accuracy

The fact that SeqTrim apparently provides the shortest sequences could be explained by over-trimming. Testing against some "gold-standards" was therefore conducted to determine trimming accuracy. A set of 100 artificially obtained sequences of 312 nt long from *P. pinaster *genomic DNA were enlarged with known vector and/or adaptor sequence at the 5'-end of the insert, and nothing and/or poly-A and/or adaptor and/or vector sequence at the 3'-end (headers of Table 1). Overall, those datasets of artificial sequences simulate 800 DNA cloning events into *Bam*HI-*Hin*dIII cloning sites of pBlueScript-FL with or without adaptors, and having or not reached the 3' cloning vector in the sequencing process. They simulate different cloning events handled by SeqTrim with a precise knowledge of insert start and end points. Hence, accuracy of SeqTrim trimming can be examined, except trimming based on quality values (QV's).

**Table 1:** SeqTrim accuracy evaluated with artificial sequences

	Vu+I			Vu+I+Vd		
	**Expected**	**Obs (enz)**	**Obs (-enz)**	**Expected**	**Obs (enz)**	**Obs (-enz)**

Sequence size	362			412		
Insert length	312	311.71	311.71	312	310.7	311.51
Insert-start point	51	51.18	51.2	51	51.2	51.2
Insert-end point	362	361.9	36.91	362	360.9	361.71
Rejected		3	0		1	0
Mistakenly processed		9	0		5	0

	**Vu+I+pA**			**Vu+I+pA+Vd**		

	**Expected**	**Obs (enz)**	**Obs (-enz)**	**Expected**	**Obs (enz)**	**Obs (-enz)**

Sequence size	392			442		
Insert length	312	311.32	311.33	312	310.72	311.33
Insert-start point	51	51.2	51.2	51	51.2	51.2
Insert-end point	362	361.52	361.53	362	360.92	361.53
Rejected		1	0		1	0
Mistakenly processed		3	0		5	0

	**Vu+Au+I**			**Vu+Au+I+Ad+Vd**		

	**Expected**	**Obs (enz)**	**Obs (-enz)**	**Expected**	**Obs (enz)**	**Obs (-enz)**

Sequence size	375			438		
Insert length	312	311.9	311.91	312	311.9	311.91
Insert-start point	64	64	64	64	64	64
Insert-end point	375	374.9	374.91	375	374.9	374.91
Rejected		2	0		0	0
Mistakenly processed		6	0		0	0

	**Vu+Au+I+pA**			**Vu+Au+I+pA+Ad+Vd**		

	**Expected**	**Obs (enz)**	**Obs (-enz)**	**Expected**	**Obs (enz)**	**Obs (-enz)**

Sequence size	405			468		
Insert length	312	311.53	311.53	312	311.53	311.53
Insert-start point	64	64	64	64	64	64
Insert-end point	375	374.53	374.53	375	374.53	374.53
Rejected		0	0		0	0
Mistakenly processed		0	0		0	0

Vu, 50 nucleotides preceding the *Bam*HI restriction site of pBlueScript-FL. Vd, 50 nucleotides following the *Hin*dIII restriction site of pBlueScript-FL. I, a fragment of 312 nucleotides from *Pinus pinaster *genomic DNA. pA, poly-A tail of 30 A's. Au, upstream 5'-adaptor, containing the sequence GATCCGTTGCTGTCGTCG. Ad, downstream 3'-adaptor, containing the sequence CGGCCGCGTCGACAAGCT. 'Expected' corresponds to theoretical mean values for each set of artificial sequences. 'Obs (enz)' are the mean values obtained using SeqTrim with the cloning restriction sites specified. 'Obs (-enz)' are the mean values obtained using SeqTrim with no cloning restriction site specified.

Launching SeqTrim with the corresponding parameters and restriction sites provided results shown in Table 1, columns 'Obs (enz)'. Rejection and incorrect processing of several sequences was not unexpected since all of these corresponded to inserts containing one of the cloning restriction sites within it. Note that a real experiment would always result in inserts without cloning sites within it, except when partially digested DNA or chimeric inserts were cloned, and in actuality, finding a cloning site within an insert is a reason to discard such an insert. As restriction site use is limited to cases where the cloning vector was not identified (see above), the same sequences were analysed without specification of restriction enzymes (columns 'Obs (-enz)' in Table 1) showing that the observed mean positions and lengths were almost identical to what was expected, and that no sequence rejection occurred. A manual inspection of results revealed that the small differences found (always lesser than 2 nucleotides) corresponded to chance instances in which the first nucleotide of the insert is the same as that following the *Bam*HI site or preceding the *Hin*dIII site of the cloning vector. In the cases were the last insert nucleotide is an A, manual inspection revealed that it was removed with the poly-A tail. In conclusion, precision of SeqTrim clips is virtually guaranteed and no over-trimming is found.

### Performance with high-throughput reads

**Figure 4:**
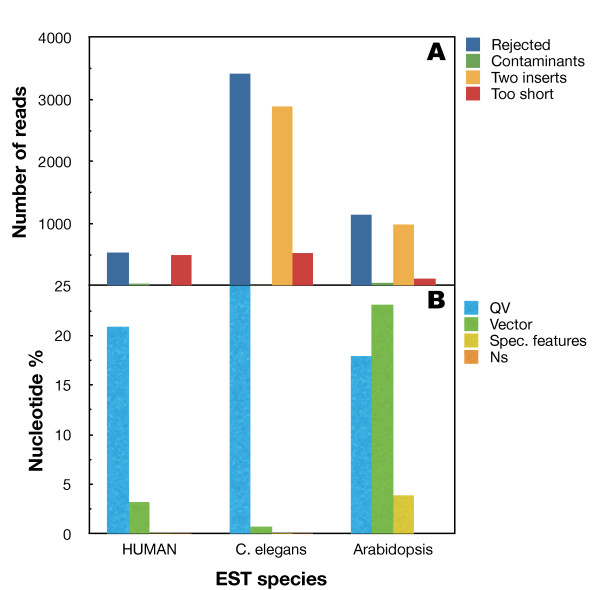
**Analysis of 30,893 ESTs from three different species**. 10,000 ESTs from the plant *Arabidopsis thaliana*, 10,244 ESTs from the worm *Caenorhabditis elegans *and 10,649 ESTs from human were clipped with SeqTrim. A, SeqTrim rejections grouped by rationale. B, percentage of nucleotides belonging to the four main SeqTrim classifications of trimming types. Rejected reads were excluded from calculations.

Sequencing projects have become a true high-throughput process with the advent of next-generation sequencing. It is of interest to test if very large-scale sequencing approaches can be processed using SeqTrim with the same previously described precision. Thus, 30,893 random reads from various different organisms from the NCBI Trace Archive were selected (Fig. 4), although no information was found regarding the pre-processor used to obtain the final clips. The first remarkable finding is that SeqTrim rejected 3418 reads in the worm *Caenorhabditis elegans *and 1146 in the plant *Arabidopsis thaliana *(Fig. 4A) mainly due to the presence of two inserts in the sequence. The surprisingly high number of rejected reads in the worm is explained by the low quality sequences considered in the dataset. Since human sequences were annotated as 're-sequencing', they were treated as genomic DNA and only 534 reads were rejected; since no restriction site was defined in SeqTrim parameters because the repository does not mention it, no read will be rejected by reason of having two inserts. Another significant aspect is that some sequence reads were assessed as being too short to be useful. Only a few reads (34 in humans, 3 in the worm and 45 in the plant) contained contaminant sequences (sequences that do not belong to the nuclear material of the analysed organism). It is clear that the *C. elegans *reads do not have the same standard of quality as the others. It can be also inferred that SeqTrim is able to detect cloning or sequencing artefacts that would not provide useful sequence for researchers in any kind of sequencing approach.

Concerning trimmed nucleotides, Fig. 4B shows the distribution of those removed due to low QV or because they were N's, cloning vector, or a specialised feature. The reads from the three species contain a high number of nucleotides discarded for having a low QV (from 17.9% in *A. thaliana *to 25.5% in *C. elegans*). These high values can be explained by the fact that the SeqTrim QV cutoff is more stringent than that of other pre-processors, although stringency can be changed in the parameters panel (see above). Additionally, human and *C. elegans *reads contain very little vector sequence (3.2% and 0.7%, respectively) while those from *A. thaliana *contain 23.1%. The specialised features are present at significant levels in *A. thaliana *ESTs, but not in the other two species. Is it important to note that, although being at low numbers, only the *A. thaliana *reads lack N's. As a consequence of trimming, 71.2% of nucleotides correspond to insert in humans, while 40.4% are insert in *C. elegans *and 43.9% in *A. thaliana*, in contrast to figures of 74.7%, 66.7% and 48.1%, respectively, that were obtained from the provided metadata.

A comparison of SeqTrim results of these sequences with the final clips reported at the NCBI was then carried out. This comparison was focused on variations of the start position and insert length since it is the only affordable comparison regarding NCBI metadata (unfortunately, only a few reads had specified which part of the sequence was removed by QV or which part is vector). The final insert in 2510 reads of *A. thaliana *and in 686 reads of human start at almost the same position as described in the database (below ± 5 nt), but none in the worm. The reason is that most *C. elegans *reads contain long stretches of cloning vector that were not removed or reported by researchers. On the other hand, 4428 reads in *A. thaliana*, 4699 in *C. elegans *and 1135 in human started in quite different positions (beyond ± 25 nt). Finally, 1916 reads in *A. thaliana*, 2127 in *C. elegans *and 8294 in human differ slightly from database reports. In any case, SeqTrim always reported shorter inserts than the database (with mean differences ranging from 1 to 318 nt). Visual inspection of the most divergent results showed that the major differences are due to more accurate localisation of the cloning vector, more stringent QV cutoff, and removal of N's. In conclusion, SeqTrim is able to analyse high-throughput Sanger sequences providing a final set of inserts of high quality and unlikely to have contaminant sequences. Moreover, it seems to outperform the software used for pre-processing the NCBI-published sequences.

## Discussion

Even if there are many DNA pre-processing algorithms in the bioinformatics literature, getting them to work correctly may be very difficult, and getting them to process high-throughput data requires an extra programming effort, to enable consideration of unlikely special cases that appear when handling large amounts of sequences or when data quality are very low [7]. Moreover, the arrival of next-generation sequencing with new experimental approaches and slightly different output format also reinforces the requirement for new software for pre-processing in a reasonable time period. Collaboration between computer scientists and biologists for SeqTrim development has permitted successful implementation of a theoretical design for a bioinformatic solution for these types of problems, and anticipated future problems.

The use of sequence pre-processors is expected to be in a pipeline with other programs [4,12,17,18]. Sometimes, constructing a pipeline is not easy, mainly due to input/output compatibility or other program peculiarities [19]. This has been considered in SeqTrim, since its flexibility regarding input and output formats contrasts with other sequence pre-processors that admit only one single type of sequence file: SeqClean, ESTPass or ESTPrep accept FASTA sequences while Lucy and pregap4 accept chromatograms. None accepts FASTA sequences plus qualities as SeqTrim does. Concerning the output compatibility, saving final sequences as trimmed or masked sequences enables the possibility if including SeqTrim in other known workflows such as phred/crossmatch/repeatmasker/phrap, or EST2UNI [19]. Moreover, most sequence pre-processors must be used only as command line programs (SeqClean, ESTPrep, TrimSeq, phred/crossmatch, Figaro), only as Web pages (VecScreen, EMBVec Query) or as command line and a GUI interface (Lucy, pregap4). No Web site is devoted exclusively to pre-processing, since they were included in more general pipelines (for example EGAssembler [18], ESTPass [11] or ESTExplorer [12]). However, SeqTrim usage was designed to be affordable by any type of user, by means of its Web interface or as a standalone application. Accordingly, the command line version is able to cope with high-throughput sequencing data while the Web interface is limited to a few thousand sequences, due to browser capabilities and the number of simultaneous connection. Nevertheless, taking into account that most SeqTrim users would be laboratory scientists who wish to pre-process their own data in order to determine how accurately their experiment were carried out, the coloured output for differentiation of trimmed regions in each sequence (Fig. 2) will facilitate interpretation of results as well as a comparison between cleaned and original sequences. In contrast to Lucy, original and cleaned sequences are on the same string, instead of two synchronised scrolling panels, to enable a first-look analysis.

Unlike other equivalent software, installation of SeqTrim does not require special skills, because there is nothing to compile, and only requires installation of freely available NCBI-BLAST, bioperl libraries, phred (only for processing chromatograms) and RepeatMasker (optional step). Configuration files and databases provided with SeqTrim can be customised, although most parameters (except restriction sites and adaptors) will never require change. In fact, SeqTrim offers more customisation possibilities than SeqClean or ESTPrep, and nearly as many as PreGap4, but does not present more than the overwhelming forty parameters that can be modified in Lucy [8]. Concerning the final performance, this study clearly demonstrated that usage of SeqTrim has significantly reduced the time and complexity involved in a number of gene discovery projects while increasing reliability (Figs. 3 and 4). Results with artificial and high-throughput sequences (Fig. 4 and Table 1) suggested that SeqTrim can handle any type of sequence read in huge numbers (considered high-throughput at least for Sanger sequencing) and provide very accurate results. Moreover, applying SeqTrim to previously published reads demonstrates that these reads should be fewer and shorter than reported (Fig. 4).

SeqTrim differential behaviour can be explained as follows: (i) unlike other pre-processors, SeqTrim is designed to remove adaptor sequences as is; note that SeqClean and ESTPrep are able to remove adaptors provided that they are included in the contamination database, which is not an easy task for unskilled users. (ii) Low quality sequence removal is more restrictive to improve the subsequent assembling procedures (Fig. 4); although this makes SeqTrim able to render the shortest sequences and a high rate of rejection, trimmed sequences produce less error-prone contigs when assembled (Fernández-Pozo, unpublished results). However, parameters that affect this behaviour are configurable by users. (iii) SeqTrim is able to remove chimeric inserts (Fig. 4)--it should be noted that the only pipeline that proclaims to be able to remove double inserts is ESTPass [11] but, in our hands, it marks as chimeric EST sequences that are not. (iv) Inserts are located by the concurrence of vector, adaptor and restriction site sequences instead of only one criterion, usually cloning sites [4], that can have experimental or base-calling errors and are too short to provide certainty. Accordingly, Table 1 shows that the presence of vector and adaptors produces slightly more accurate results than vector alone. (v) In contrast to most pre-processing pipelines, low quality sequences are not removed in early pre-processing since location of vector, adaptor, restriction site, and poly-A/T are more reliable using crude sequences (Fig. 2). Furthermore, starting with low quality removal may obliterate key information like sequence orientation or presence of poly-A or poly-T tails. In contrast, the number of sequences rejected due to two inserts can increase, as long A or T tails can be found in low quality reads. These facts can be interpreted as concomitant poly-A and poly-T tails in the same insert, and therefore the sequence is rejected before analysing its QV, which would be the true reason for rejection. (vi) Since localisation of vector, adaptors and contaminant sequences are not dependent on perfect matches, any slippage occurring at those regions will be successfully treated, while slippage in inserts is not detected. (vii) SeqTrim is ready for handling next-generation sequencing artefacts where results are provided as sequences and qualities in independent files. Since 20,000 EST sequences will take more than one hour and since a single run of a 454 machine can generate 5 million sequences (which could take approximately 5 days to execute), future efforts for SeqTrim improvements will be focused on parallelisation and function optimisation in order to reduce execution times, as well as providing robustness for these huge numbers of sequence.

## Conclusions

SeqTrim is the product of years of collaboration between computer scientists and biologists at the University of Málaga and is under continuous development. It scales up for pre-processing huge sets of sequences (including next-generation sequencing of bacterial artificial chromosomes one-by-one) using large-scale parallel computers (Web version), providing a time- and cost-effective solution, and its Web interface is user-friendly. Although most parameters will never require change, SeqTrim offers sufficient customisation and can cope easily with adaptors and chimeras, as well as next-generation sequencing artefacts. Input/output features provide more flexibility than other pre-processors for integration in workflows with other programs or in existing ones. The coloured output facilitates differentiation of trimmed regions in each sequence and paves the way for result interpretation as well as comparison between cleaned and original sequences, since they are on the same string. Accurateness and reliability of the final sequence clip obtained by SeqTrim have been clearly demonstrated.

## Availability and requirements

**Project name **SeqTrim. No license or account needed.

**Availability **http://www.scbi.uma.es/seqtrim. Source code is directly available from the Web page. It does not include the third party software required. It also includes the vector and contaminant databases used in this work.

**Operating systems **Platform-independent (both Web and command line application).

**Programming languages **Perl for algorithm; Javascript and HTML for Web interface.

**Other requirements **Web browser supporting JavaScript (preferably Mozilla Firefox or Apple's Safari). For the command line version, the computer should have installed BioPerl http://www.bioperl.org/wiki/Getting BioPerl, NCBI-Blast http://blast.ncbi.nlm.nih.gov/Blast.cgi?CMD=Web&PAGE_TYPE=BlastDocs&DOC_TYPE=Download, phred http://www.phrap.org/phredphrapconsed.html only in case of using SeqTrim with chromatograms, and optionally RepeatMasker http://www.repeatmasker.org/. Execution of RepeatMasker requires further installation of WU-Blast http://blast.advbiocomp.com/licensing/ or cross_match http://www.phrap.org/phredphrapconsed.html.

## Authors' contributions

JF coded in PERL the software as a command line. AJL designed and tested the Web interface. NFP tested the command line and Web interface with experimentally- and artificially-derived sequences. FRC obtained the chromatograms and next-generation sequences for testing SeqTrim, and verified by hand the output reliability. GPT connected the Web interface with super-computation capabilities at Málaga University. MGC designed the software, took into account its compatibility, and helped FRC in the manual inspection of trimmed sequences. All authors read and approved the final manuscript.

## Appendix

### Pseudocode of functions and algorithms developed specifically for SeqTrim

   function findPolyATs(sequence, minLen, poly_type)

      if found (at least minLen/2) AorT

            (then [0..3] other)

            (and at least minLen/2 AorT)

      {

         expand the region with {(at least minLen/4) AorT

            (then [0..2] other)

            (and at least minLen/4 AorT)}

            at both sides up to 3 bases from each each end

         Ns flanking the region are removed

         wasThereASecondpolyTorA = Look for a second polyAorT after this one

      }

      return [start_point, length, wasThereASecondpolyTorA]

   function FindContaminantSeqs(seq)

      Run BLASTN against local contaminant database

      Ignore shorter than a minimal contamination length *# *defined by a user parameter if user gives a genus

         Compare the contaminants found with the genus as it was not contaminant

      Build up a list with the names of all the real contaminants

      If the contaminant region is longer than admitted

         the sequence is rejected.

      *# *Look for adaptors as a key for chimeric inserts

      Run BLAST2SEQ against adaptors (5' and 3' ends)

      if found but the distance is longer than the own adaptor length

         the seq is rejected

   function Look_for_artefacts(seq)

      if "GCGGGG" or "CCCCGC" found at 5' or 3' end

         Delete it

      Clean up from extreme Ns and Xs

      If is cDNA, and forward read, remove first Ts and last As (if more than 3)

      If is cDNA, and backward read, remove first As and last Ts (if more than 3)

   function LowComplexityMasking(s)

      *# *masking repeats

      Run RepeatMasker using WU-BLAST

      Analyse the result file getting [ID, beg, end, what, class]

      Mask with Xs all regions found in each ID

      *# *masking dust

      Look for all but Xs repeated at least 5 times

      Mask them with Xs
